# Elemental composition, structure and amount of contact between *Xanthoria parietina* symbionts

**DOI:** 10.1038/s41598-026-68076-7

**Published:** 2026-08-31

**Authors:** Andreas Beck, Christian Bayer, Rafaela Debastiani, Tim Schubert, Andreas Gröger, Bernhard Ruthensteiner

**Affiliations:** 1 Department of Lichenology and Bryology, Botanische Staatssammlung München, SNSB-BSM, 80638 Munich, Germany; 2 Systematics, Biodiversity and Evolution of Plants, Faculty of Biology, Ludwig-Maximilians-Universität München, 80638 Munich, Germany; 3 GeoBio-Center, Ludwig-Maximilians-Universität München, 80333 Munich, Germany; 4 Quantitative Methods, Aalen University of Applied Sciences, 73430 Aalen, Germany; 5 Institute of Nanotechnology (INT), Karlsruhe Institute of Technology (KIT), 76131 Karlsruhe, Germany; 6 Karlsruhe Nano Micro Facility (KNMFi), Karlsruhe Institute of Technology (KIT), 76344 Eggenstein-Leopoldshafen, Germany; 7 Helmholtz Institute Freiberg for Resource Technology (HIF), Helmholtz Zentrum Dresden Rossendorf (HZDR), Chemnitzer Strasse 40, Freiberg, Germany; 8 Materials Research Institute Aalen, Aalen University of Applied Sciences, 73430 Aalen, Germany; 9 Botanischer Garten München-Nymphenburg, SNSB-BGM, 80638 Munich, Germany; 10 Zoologische Staatssammlung München, SNSB-ZSM, 81247 Munich, Germany

**Keywords:** Ecology, Ecology, Microbiology, Plant sciences

## Abstract

As prominent examples for symbiotic interactions, the major part of lichens is built up by two different organisms, a photosynthetically active alga (photobiont) and a heterotrophic fungus (mycobiont). Given the hydrophobic coating of lichen symbionts in the lichen thallus by hydrophobins, the area of direct cell cell contact is of prime importance for the transfer of nutrients and signalling compounds between the symbionts. Genomic analyses reported cell wall remodelling during symbiont interaction. However, no detailed investigation of lichen symbiont interaction zone is available so far. Using Energy-dispersive X-ray spectroscopy (EDS), we show that elemental composition of both symbiont cell walls differs when in contact to the symbiont as opposed to those cell wall areas without contact. Both, nitrogen and sulfur content of cell walls, differ significantly in areas of symbiont cell contacts as compared to non-contact zones. NanoCT imaging revealed that about one fourth of the photosymbiont cell wall is in contact to mycobiont hyphae, and fungal cells have a significantly increased volume if in contact to photobiont cells. We provide a sub µm 3D model of the lichen *Xanthoria parietina* using nanoCT imaging to foster understanding of the symbiont interaction zone.

**Supplementary Information:**

The online version contains supplementary material available at https://doi.org/10.1038/s41598-026-68076-7.

## Introduction

Lichens are originally defined as symbiotic entities in which fungi and algae interact and form a new structure – the lichen thallus^1–4^. These fascinating symbiotic units formed early in earth history, with fossils dating back to 415 million years ago^5^ and are such prominent examples, that they were mentioned in the definition of the term symbiosis by DeBary^6^. Comparing this old symbiotic interaction with other fungal plant interactions, e.g. mycorrhiza, may help to explain and improve the latter ones. Consequently, the lichen symbiosis has received increasing attention recently, due to advances in omics techniques such as genomics and metabolomics. Genomics is uncovering also the minor constituents of the lichens leading to discussions on the definition of lichens^7–10^. Metabolomic studies^11–14^ elucidated the interactions of the symbionts on the bases of the metabolites produced and provided important insights into these interactions. Likewise, metabolomics studies helped to elucidate the symbiotic relationship of the symbionts^15–17^. Advanced imaging techniques were also applied, albeit not extensively. The distribution of endolichenic bacteria and endolichenic fungi residing within lichens has been analysed^18–20^, extending analyses beyond the primary symbiotic partners inherent to the lichen thallus. Knowledge on the fine structure of the lichen thallus had been advanced considerably by Honegger and co-workers using mainly electron microscopy^21^. Consequently, there is reasonably good knowledge of the 2D-interaction zone of lichen fungi and algae, but 3D information is lacking. Using µCT techniques, the volume of cephalodia – structures on/in the lichen thallus extending the symbiotic relationship to cyanobacteria as a third partner – were quantified for *Peltigera leucophlebia* (Nyl.) Gyeln. and *Lobaria pulmonaria* (L.) Hoffm.^22^. The external cephalodia of the former comprised up to 0.97% of the thallus volume, the internal cephalodia of the latter amounted to 0.73%. Nevertheless, resolution was not yet sufficient to resolve the interaction zone of lichen fungi and lichen algae in three dimensions.

The interaction zone between the lichen symbionts is of high interest to understand their interplay. This is the region were nutrient transfer between the symbionts takes place. So far, only the transfer of carbohydrates has been verified, while nitrogen transfer is still elusive^23,24^. On the 2D level, it has been reported that morphological changes occur upon symbiont interaction and that fungal cells develop a swollen hyphal tip and an appressorium/haustorium-like structure when growing in contact to photobiont cells, presumably increasing interaction surface and facilitating nutrient transfer^25–27^.

However, 3D representation is preferable over 2D because it more accurately captures the symbiont interaction zone. This superiority stems from its ability to depict depth, volume, and perspective, elements absent in the flat, limited world of two dimensions. It offers a richer, more detailed, and consequently, more informative scientific visualization. Thus, we intended to use a high resolution sub µm 3D data set based on a nanoCT (nCT) scan to measure fungal cells at different distances from the photobionts and determine the amount of interacting cell surface.

Cell walls are still present in the symbiont cell-cell interactions in lichens^28^. These are known to serve many functions modulating important processes, such as interactions with neighbouring cells or the surrounding environment as well as growth and are not static structures, but are highly dynamic^29^. Cell wall remodelling at fungal-algal contact sites has been reported as one of the first steps in the establishment of the lichen symbiosis^30^. But the likely resulting different composition of the cell wall when in symbiont contact as compared to solitary growth has not yet been addressed. Furthermore, cell wall remodelling is known to occur in *Trebouxia* photobionts upon cyclic desiccation–rehydration^31^. Cell wall components are also thought to be involved in the desiccation tolerance of lichens and their photobionts^32^. In the *Trebouxia* photobiont cell wall β−3-linked rhamnogalactofuranan, KOH-soluble β-glucans, sulfated polysaccharides, and glycosylated proteins have been detected^31,32^. Structural proteins have been reported in the cell wall of *Trebouxia* photobionts^33^, as well as hydrolases and proteins involved in ROS scavenging and cell-cell interaction in the extracellular polymers^31^. In the true fungi, like the *Xanthoria parietina* mycobiont, the inner layer of the cell wall is primarily composed of chitin and linear glucans and gives stability to the cell^29,34^. Also the β−1,3/1,4-glucan lichenin has been demonstrated in lichen mycobionts^3^. Additionally, fungal cell walls contain varying amounts of homo- or heteropolysaccharides, which can be components of glycoproteins^35,36^. Energy dispersive X-ray spectroscopy (EDS) is a very versatile analytical technique available for materials analysis^37^. In EDS electron microscopy is coupled to elemental spectroscopy, rendering information on the elemental composition of the analysed sample, widely used in biomedical research^38^. In lichens it has been widely used to study metal uptake^39,40^, as well as biomineralization^41^, and biomonitoring airborne heavy metal pollution^42^. Nevertheless, details like symbiont cell wall composition have not yet been investigated.

Given the high importance of the interaction zone for the lichen symbiosis, we chose to investigate the elemental composition of the cell walls of the symbionts with and without contact to the symbiotic partner using energy-dispersive X-ray spectroscopy (EDS). In order to elucidate the three-dimensional structure and the amount of cell-cell contact in the interaction zone, we chose the widely distributed yellow wall lichen, *Xanthoria parietina* (L.) Beltr., whose populations have increased enormously during the last decades due to its resistance to air pollution, especially oxidized and reduced forms of nitrogen arising from combustion processes and agriculture, respectively^43^. It has been shown before to be composed of the fungus *Xanthoria parietina* (L.) Th. Fr. and *Trebouxia decolorans* Ahmadjian in southern Bavaria^24^. Moreover, it is one of the best investigated lichens and frequently used as a model for the lichen symbiosis^24,44–46^.

## Results

### Cell wall composition varies between symbiont contact and solitary condition

**Fig. 1:**
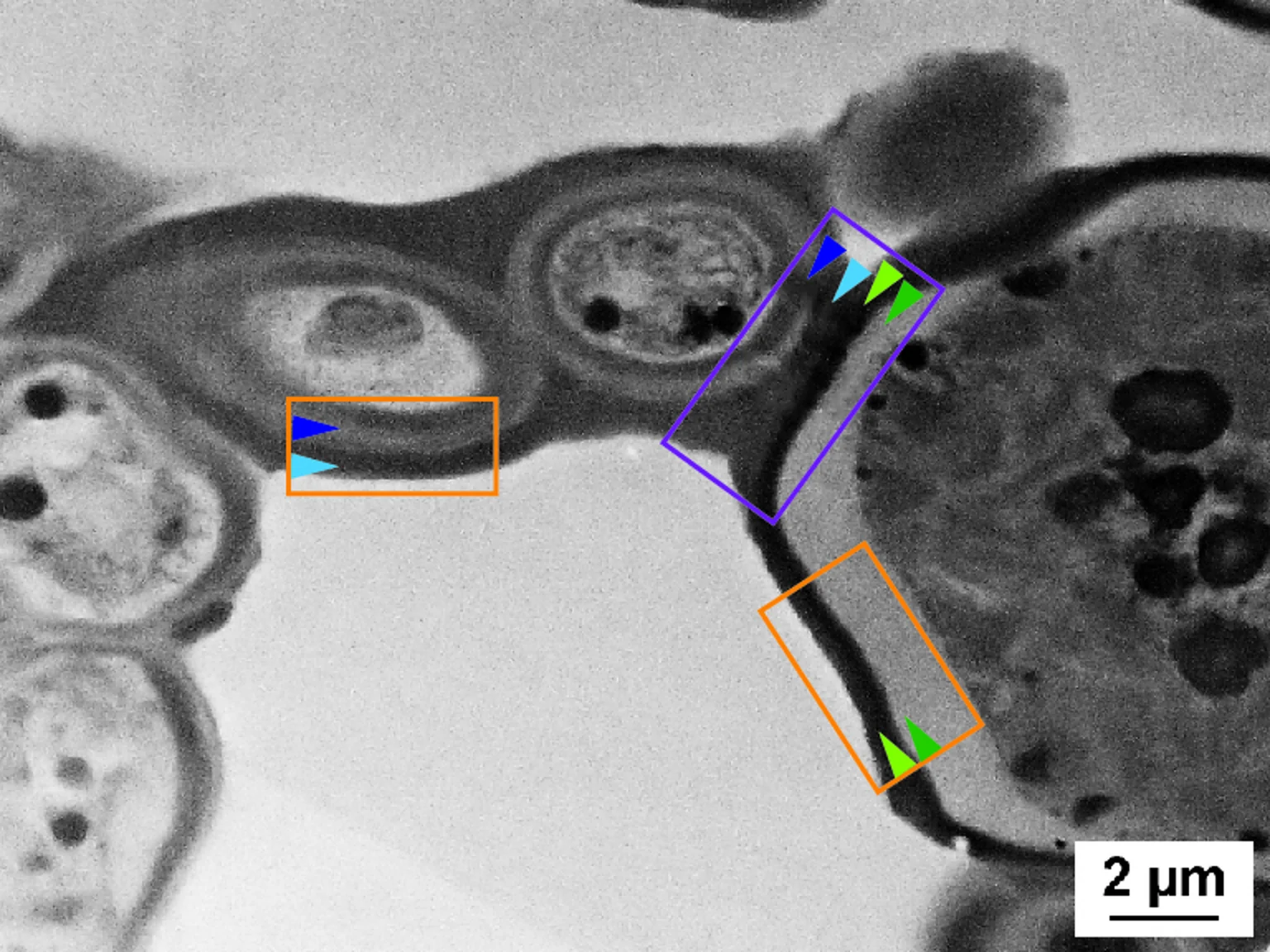
FIB-SEM image of the algal-fungal contact zone. Note the layered structure of the cell walls. Areas for measurements are indicated by the lilac (symbiont contact) and orange (solitary cell walls) rectangles. Algal and fungal cell wall parts are indicated by green and blue arrows, respectively, with the lighter colour indicating the outer, and the darker colour indicating the inner cell wall parts.

**Fig. 2:**
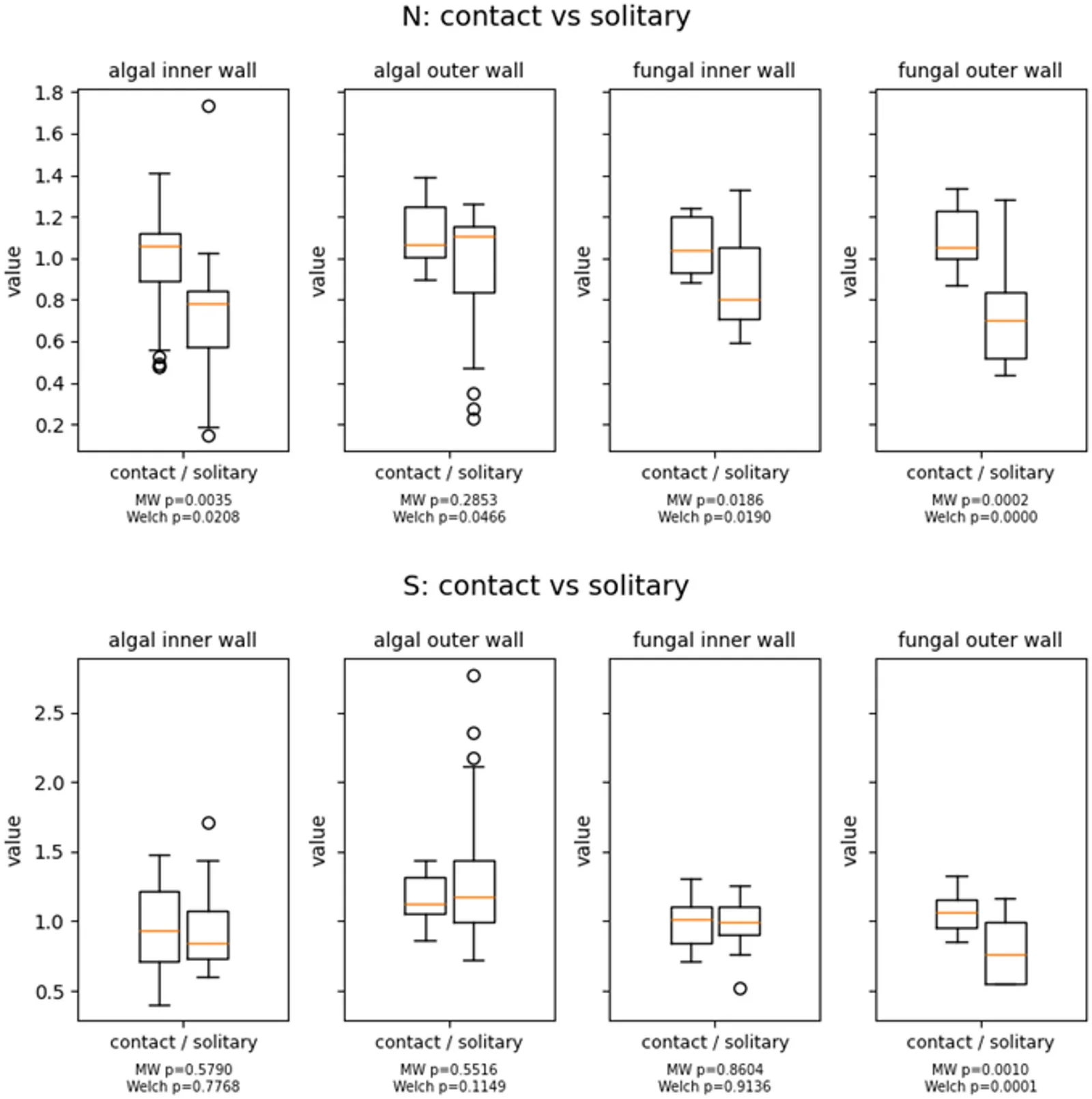
Elemental composition of lichen symbiont cell walls as revealed by EDS analyses. The cell walls have been subdivided into the algal inner wall, the algal outer wall, the fungal outer wall and the fungal inner wall for both: areas of cell-cell contact (left boxplot) and areas of the cell growing solitary (right boxplot). Refer to Fig. 1 for more detailed localization. Upper part: nitrogen (N), lower part: sulphur (S). Below the boxes the results of the Mann Whitney U Test (MW) and the Welch’s t-test (Welch) are given. Value refers to the harmonized percentage of the respective spectrum (see material and methods for details).

The cell walls of contacting symbionts (*Xanthoria parietina* (L.) Th. Fr. and *Trebouxia decolorans* Ahmadjian) can be divided in four parts: algal inner wall (bright), algal outer wall (dark), fungal outer wall (dark), fungal inner wall (bright) (Fig. 1, lilac rectangle). Elemental analyses using Energy-dispersive X-ray spectroscopy (EDS, Ultim Extreme Detector, Oxford Instruments GmbH) in a Scanning Electron Microscope (Crossbeam 540, Carl Zeiss Microscopy GmbH) revealed, that nitrogen (N) is enriched in the symbiont contact zone as compared to the solitary condition for all cell wall layers (Figs. 1 and 2). Harmonized mean values were between 1.12 and 0.97 for the contact area and between 0.94 and 0.72 for the area without symbiont contact. For sulfur (S) values were more similar differing mainly in the fungal outer cell wall. Harmonized mean values were higher when in symbiont contact for the inner algal and outer fungal cell wall (ranging between 1.06 and 0.96 versus 0.93 and 0.79 when solitary), while the reverse was true for the symbiont contact for the outer algal and inner fungal cell wall (ranging between 1.15 and 0.99 versus 1.37 and 0.99 when solitary). These differences were found to be significantly supported in both, the Mann Whitney U Test and the Welch’s t-test, for the N content of the algal inner wall as well as the fungal inner and outer wall and for the S content of the fungal outer cell wall (Fig. 2).

### Sub µm 3D-model of the symbiont interaction zone

**Fig. 3:**
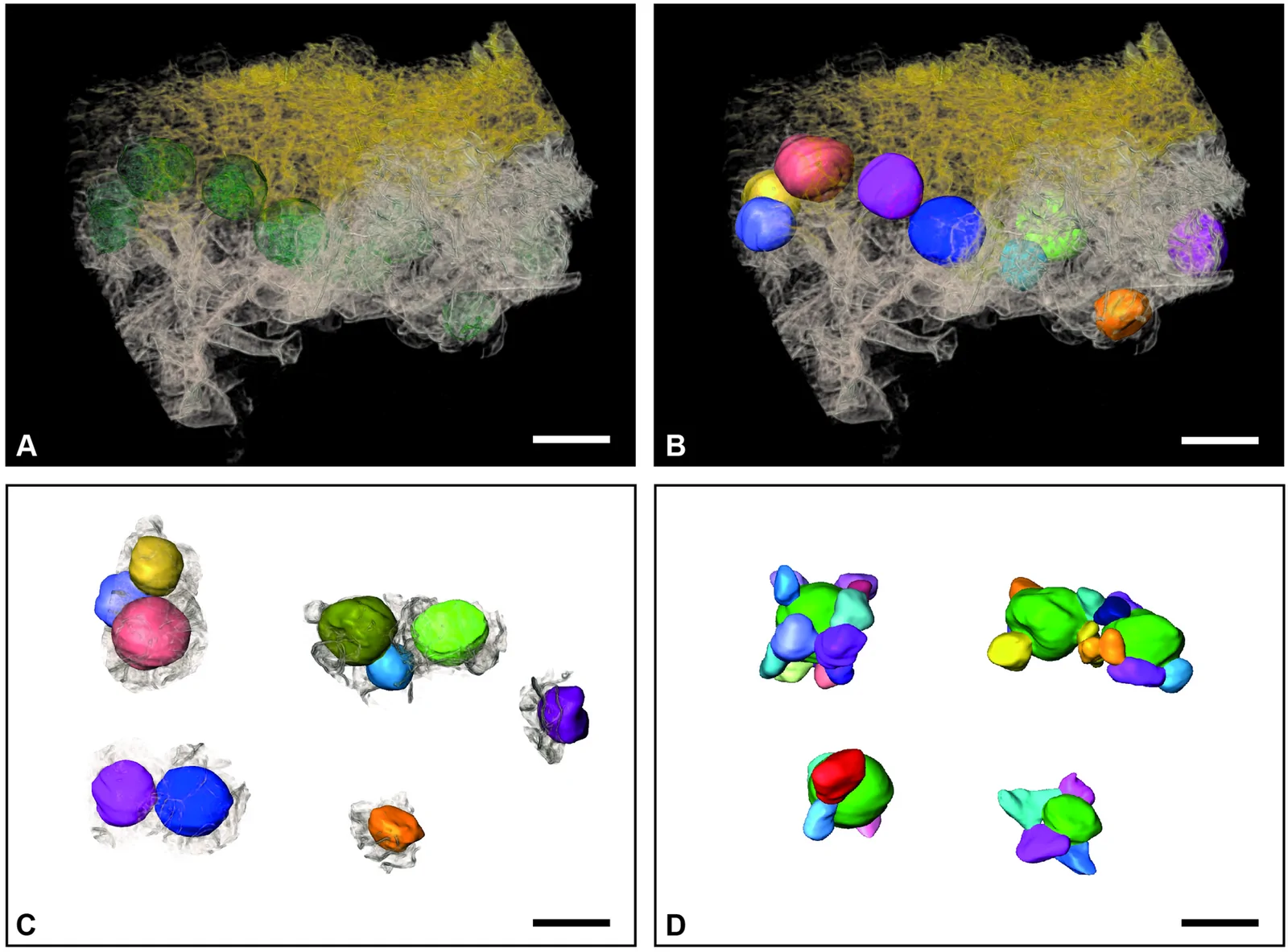
nCT model of *Xanthoria parietina*. (**A**) Model including the symbiont interaction zone and the cortex. Algae are coloured in green, fungal hyphae in grey and the cortical hyphae in yellow. (**B**) Same model as in A, but with colour coded individual algal cells. (**C**) Colour coded individual algal cells as in B, including the area around them up to 1.5 times the diameter of each cell. (**D**) Individual algal cells (coloured in green) with the contacting fungal hyphae as used for the calculation of the contact area. Scale bar: 10 μm.

We provide the first 3D model of the interaction zone between lichen fungi and algae. Using a sequence of optics in the nanoCT, we could achieve a voxel size of 0.127 μm. The Zernike phase contrast mode allowed the visualization of the structures through edge enhancement. 2D representations are depicted in Fig. 3, while an interactive 3D model is presented as Supporting Information Fig. S1.

### Cell sizes

**Fig. 4:**
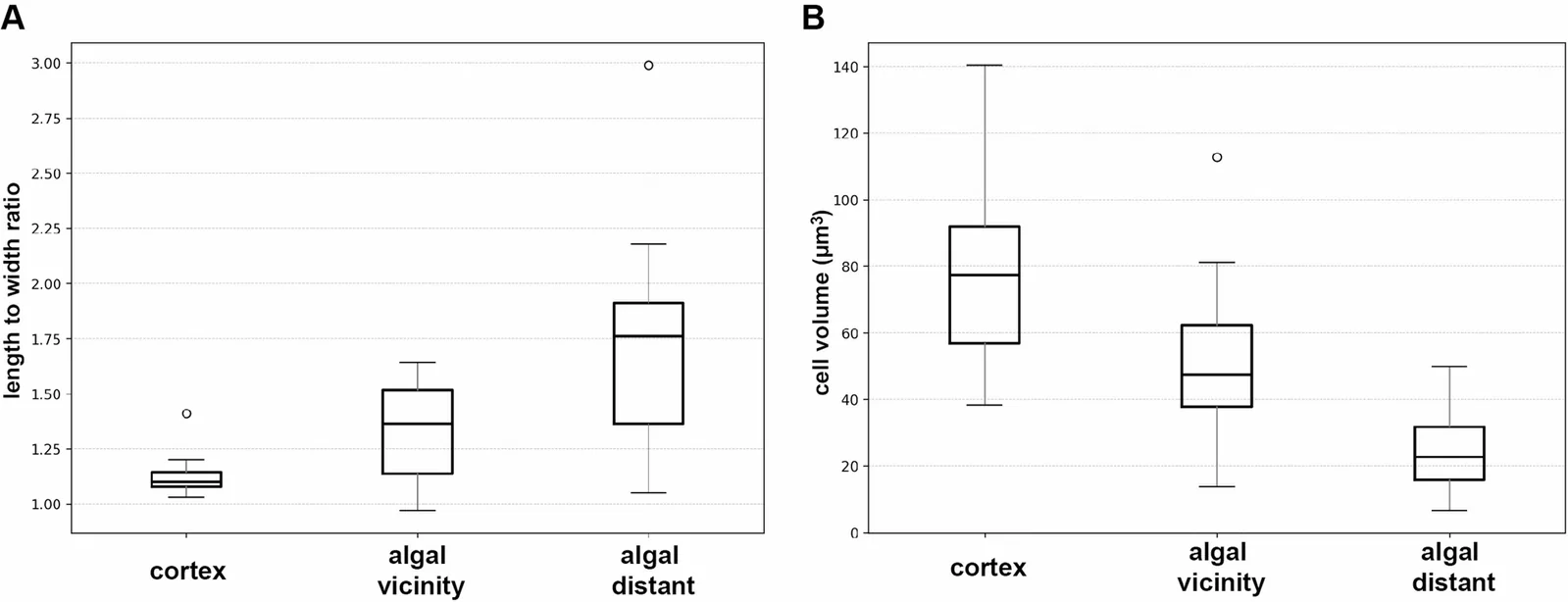
Cell dimensions of fungal cells belonging to the cortex, close to photobiont cells (up to 1.5 times the diameter of the algal cell) and more distant to the algal cells as inferred from the nCT model of *Xanthoria parietina*. (**A**) Length to width ratio. (**B**) Cell volume.

Fungal cells in the vicinity of photobiont cells (cells closer than 1.5 times the diameter of the algal cell) varied in length from 4.15 to 6.92 μm (mean 5.85 μm, standard deviation 1.1 μm) and in width from 3.38 to 4.88 μm (mean 4.45 μm, standard deviation 0.8 μm). Mean length to width ratio was 1.32 μm with a standard deviation of 0.2 μm (Fig. 4A). The volume of the cells ranged from 13.83 to 112.81 µm^3^, with a mean of 52.90 µm^3^ and standard deviation of 24.68 µm^3^ (Fig. 4B). Fungal cells more distant to photobiont cells than 1.5 times the diameter of the algal cell proved to be elongated varying in length from 4.49 to 8.13 μm (mean 5.92 μm, standard deviation 1.1 μm) and in width from 2.29 to 5.35 μm (mean 3.65 μm, standard deviation 1.0 μm). Mean length to width ratio was 1.72 μm with a standard deviation of 0.51 μm. The volume of the cells ranged from 6.61 to 49.88 µm^3^, with a mean of 26.56 µm^3^ and standard deviation of 13.63 µm^3^. Cortex cells varied in length from 4.72 to 7.61 μm (mean 5.92 μm, standard deviation 0.8 μm) and in width from 4.00 to 7.08 μm (mean 5.27 μm, standard deviation 0.9 μm). Mean length to width ratio was 1.13 μm with a standard deviation of 0.09 μm. The volume of the cells ranged from 38.35 to 140.40 µm^3^, with a mean of 77.59 µm^3^ and standard deviation of 28.73 µm^3^. Statistical analysis revealed the suitability of ANOVA for the volume dataset, only, and consequently the parameter free Kruskal-Wallis test was used for the mean length to width ratio dataset. All mean values were found to differ significantly from each other.

The nCT model enabled to determination of the amount of photobiont cell surface involved in the contact with fungal hyphae for the first time. Between 13% (lower left algal cell in Fig. 3D) and 35% (upper left algal cell in Fig. 3D) of the algal surface neighboured to fungal cells, resulting in a mean value of 22.5% with 8% standard deviation.

## Discussion

Morphological studies of lichens have been challenging, and µCT studies used iodine as contrast-enhanced agent in X-ray computed tomography^22^. Taking advantage of new possibilities of high-resolution 3D CT systems using the Zernike phase contrast mode, we were able to obtain a high-resolution 3D model without the application of contrast-enhancing agents. A voxel size of 0.127 μm theoretically allows a true spatial resolution of 0.32 μm (2.5 times the voxel size) and therefore routine detection of structures of this size. We were therefore able to measure cell dimensions in the symbiont interaction zone of the studied specimen of the lichen *Xanthoria parietina* very accurately, identifying not only length and width of the interacting cells accurately, but also the volume. We endorse the previously observed swelling of fungal cells in close proximity (closer than 1.5x the diameter of the algal cell) to algal cells^25,27^ and show for the first time, that this swelling leads to almost a doubling of the mean cell volume of fungal cells in close proximity to algal cells as compared to the more distant ones. The largest fungal cell volumes could be observed in the almost isodiametric cortical cells, with volumes three times as big as the mean cell volume of fungal cells in close proximity to algal cells. Our 3D analysis also revealed that almost one fourth of the photobiont cell surface is typically in contact with fungal hyphae and thus involved in the direct exchange of metabolites between the mayor symbionts. Nevertheless, interspecific variability in these cell volumes and contact area will have to be determined in detail in follow up studies.

Overall, the work described here reveals the different elemental composition of the lichen symbiont cell walls if in contact to each other as compared to solitary growth. Both, N and S percentages are higher in the symbiont contact zone. This highlights the functional importance of the cell-cell contact zone as such, with the amount of cell-cell contact determining the extent of interaction. In the *Trebouxia* cell walls, structural and glycosylated proteins are the mayor source for nitrogen^33,47^. Sulfated polysaccharides are the mayor source for S in the algal cell walls^47^. Both, the inner and the outer algal cell wall parts are significantly enriched in N in the symbiont contact zone. This indicates a difference in structural, but probable also functional proteins, possibly involved in the symbiosis. Only the inner algal cell wall showed a higher S content, most likely related to the content of sulfated polysaccharides, which could also relate to altered cell wall properties.

The fungal inner wall largely determines shape and strength of the fungal cell^48^. This may explain why the difference in the elemental composition of the inner fungal wall is smaller as compared to the outer wall. The latter determines properties of adhesiveness, hydrophobicity, and chemical heterogeneity likely of importance in the interaction of the lichen symbionts^48^. Polysaccharides and glycoproteins composing the outer and inner cell wall layers represent fungus-specific pattern recognition molecules and antigens^49^, representing likely candidates for determining the interaction type between lichen fungi and lichen algae. Nevertheless, those studies in lichens are still in its infancy, but urgently needed to better understand the lichen symbiosis.

## Materials and methods

*Xanthoria parietina* was collected close to the park of Nymphenburg Castle, Munich, Upper Bavaria, Germany. No permission was needed to collect the sample on these public grounds. The remaining specimen is stored at the Botanische Staatssammlung München, SNSB-BSM (herbarium M) with the herbarium number M-0367645. The lichen was determined purely by morphological characters, as it has been shown before to be composed of the fungus *Xanthoria parietina* (L.) Th. Fr. and *Trebouxia decolorans* Ahmadjian in southern Bavaria^24^. The sample for nanoCT examination was processed immediately after collection. One square cm of marginal thallus was taken off with sterile tweezers and dehydrated twice in 400 µl acidified 2,2 Dimethoxypropane (DMP; Merck, Darmstadt, Germany) for 30 min. Then the DMP was replaced three times by 500 µl acetone (Merck, Darmstadt, Germany) and critical point dried using the Polaron E 3000 machine (Quorum Technologies, Sussex, UK). The sample was then placed in silica xerogel (Carl Roth GmbH, Karlsruhe, Germany) until CT scanning (one week after collection).

### NanoCT preparation and acquisition

The nanoCT imaging was carried out using the lab-based X-ray microscope Zeiss Xradia 810 Ultra (Carl Zeiss Microscopy GmbH; Oberkochen, Germany). This system uses a Cr-anode to produce a quasi-monochromatic X-ray beam with energy of 5.4 keV, a sequence of optics to achieve fields of view of 16 or 65 μm and maximum resolution of 50 nm for a pixel size of 16 nm, in absorption and Zernike phase contrast mode^50,51^. The samples were prepared for the nanoCT by gluing the lichen on the top of a graphite needle using UV glue. The *X. parietina* sample was scanned over a rotation of 180° within a field of view of 65 μm and a pixel size of 127.2 nm in Zernike phase contrast mode. 901 projections were acquired with an exposure time per projection of 60 s, resulting in a total acquisition time of 18.5 h. The reference acquisition was carried out before and after the scan and each reference corresponds to the acquisition 40 projections. The 2D projections were reconstructed using the proprietary software Zeiss Scout and Scan™ Reconstructor version 13 (Carl Zeiss Microscopy GmbH; Oberkochen, Germany; https://www.zeiss.com/microscopy/us/l/campaigns/scout-and-scan.html#download), based in filtered back projection (FBP) algorithm.

### The data visualization and segmentation

The 16 bit data sets generated by reconstruction were cropped, histogram adjusted and converted to 8 bit. Further 3D graphical procedures were conducted with Amira-Avizo 3D version 2023-2 software (Thermo Fischer Scientific, Electron Microscopy Solutions, Hillsboro, Oregon, USA; https://www.thermofisher.com/software-em-3d-vis/customerportal/download-center/amira-avizo-3d-installers/).

Segmentation was performed manually according by subjective judgment. Since in cortical cells no cell wall area can be separated from neighbouring cells, a uniformly thin area around the lumen was added as a cell area. The cell segmentations were used to create surfaces which were used for measurements. Distances were assessed using the Line tool in the Measurement module and surface area dimensions were determined using the Surface Area Volume module.

For the cortical cells and the hyphal cells distant from the algal cells, the three axes were selected so that the longest extension was determined and measured first. The other two spatial directions were measured perpendicular to this and chosen so that the largest possible value represented the width and the smaller value represented the height. For the fungal hyphal cells adjacent to the algal cells, the direction of the axes was chosen so that the length was radial to the fungal hyphal cell and the other two directions perpendicular to it gave the largest possible value (width) and the smaller value (height) perpendicular to it.

For further visualization, the Volume Rendering module was used in addition to the Surface Rendering module, to display different areas.

### EDS analysis

The lichen samples were fixed for 2 h at room temperature with 2.5% glutaraldehyde in fixative buffer (75 mmol/liter cacodylate/2 mmol/liter MgCl_2_, pH 7.0). After the lichen had been rinsed several times with the same buffer, samples were postfixed for 2 h with 1% osmium tetroxide in fixative buffer. After four washing steps with fixative buffer and distilled water the lichen sample was dehydrated in a graded series of acetone solution: 10%, 20%, 40%, 60%, 80%, and 100% (en bloc staining was carried out with 1% uranylatetate at the 20% stage). Lichen fragments were infiltrated and embedded in Spurr’s low-viscosity resin.

The chemical composition of the different cell regions was determined on a focused ion beam cross section prepared with a Zeiss Crossbeam 540 (Carl Zeiss Microscopy GmbH; Oberkochen, Germany). The cross sections where prepared with 1.5 nA beam current for minimal ion-induced damage while maintaining sufficient milling efficiency. Chemical analyses were performed using an Oxford Instruments Ultim Extreme EDS detector, optimized for high sensitivity at low beam energies. Point spectra EDS measurements were acquired at 1.5 kV and 5 kV to allow for surface-sensitive and bulk-sensitive elemental analysis. Four independent cross-sections of two thalli were measured. In each cross-section, for each measurement area five point spectra were collected for statistics. All spectra were processed using the manufacturer’s software AZtecLive version 6.3 (Oxford Instruments, High Wycombe, UK; https://nano.oxinst.com/products/aztec/aztec-6-3) applying standard background subtraction and peak deconvolution procedures.

### Statistical analysis

Calculations have been done using the packages NumPy version 2.2.0 (https://numpy.org/) and pandas version 2.2.3 (https://pandas.pydata.org/). Plots were drawn using matplotlib version 3.9.0 (https://matplotlib.org/).

For EDS analyses, it cannot be expected to get the same results – especially for light elements like C, N, and O – when the sample conditions just slightly change e.g. due to new preparation or different positions. This is due to the fact, that low‑energy K lines (< ~ 1 keV), that are present especially for light element analysis, are heavily absorbed in the sample, surface and detector. Consequently, small changes in geometry cause large intensity changes^52–54^. Standardless routines typically have larger systematic errors, with light elements are inherently more sensitive to all of these factors, even when using todays improved standardless methods^55–57^. We noticed this as well for our different cross-sections, thus we only compared the differences of the values for cell wall measurements with/without symbiont contact. To allow statistical analysis of all cross-sections together, we harmonized the measurements in-between different cross-sections by calculating the median for all measurements within one cross-section and dividing the measurements by this median (baseline harmonization). The resulting values (four cross-sections x five measurements each = 20) were combined for all eight conditions (inner algal wall with symbiont contact; inner algal wall without symbiont contact; outer algal wall with symbiont contact; outer algal wall without symbiont contact; inner fungal wall with symbiont contact; inner fungal wall without symbiont contact; outer fungal wall with symbiont contact; outer fungal wall without symbiont contact), and the four corresponding datasets (with/without symbiont contact) tested for statistical difference using the packages mentioned above.

## Supplementary Information

Below is the link to the electronic supplementary material.


Supplementary Material 1


## Data Availability

All generated data is available in the Figures, including the supplementary Figure.
