# Supplementary material for: Elemental composition, structure and amount of contact between Xanthoria parietina symbionts
Source: Sci Rep. 2026 Aug 31;16:27379. doi: 10.1038/s41598-026-68076-7 (PMC13534624; doi:10.1038/s41598-026-68076-7)

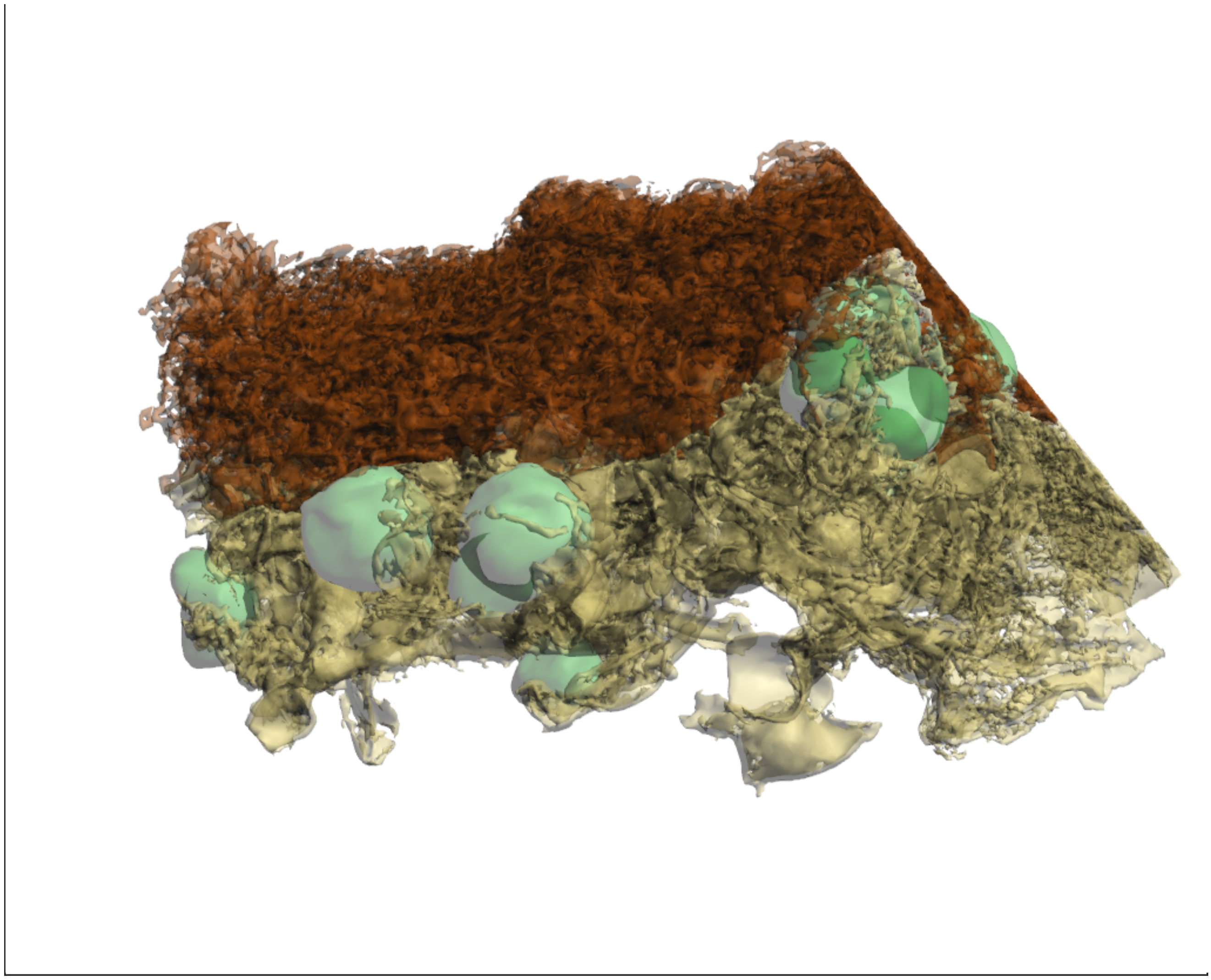

Supplement: Supplementary file 1 [file 41598_2026_68076_MOESM1_ESM.pdf]
